# Mitochondrial Calcium Transporters Mediate Sensitivity to Noise-Induced Losses of Hair Cells and Cochlear Synapses

**DOI:** 10.3389/fnmol.2018.00469

**Published:** 2019-01-08

**Authors:** Xianren Wang, Yuanping Zhu, Haishan Long, Song Pan, Hao Xiong, Qiaojun Fang, Kayla Hill, Ruosha Lai, Hu Yuan, Su-Hua Sha

**Affiliations:** ^1^Department of Pathology and Laboratory Medicine, Medical University of South Carolina, Charleston, SC, United States; ^2^Department of Otorhinolaryngology, The First Affiliated Hospital, Sun Yat-sen University, Guangzhou, China; ^3^Department of Otorhinolaryngology, Renmin Hospital of Wuhan University, Wuhan, China

**Keywords:** mitochondrial calcium uniporter (MCU), sensory hair cell, ribbon synapses, noise-exposure, auditory threshold shifts, mouse model

## Abstract

Mitochondria modulate cellular calcium homeostasis by the combined action of the mitochondrial calcium uniporter (MCU), a selective calcium entry channel, and the sodium calcium exchanger (NCLX), which extrudes calcium from mitochondria. In this study, we investigated MCU and NCLX in noise-induced hearing loss (NIHL) using adult CBA/J mice and noise-induced alterations of inner hair cell (IHC) synapses in MCU knockout mice. Following noise exposure, immunoreactivity of MCU increased in cochlear sensory hair cells of the basal turn, while immunoreactivity of NCLX decreased in a time- and exposure-dependent manner. Inhibition of MCU activity *via* MCU siRNA pretreatment or the specific pharmacological inhibitor Ru360 attenuated noise-induced loss of sensory hair cells and synaptic ribbons, wave I amplitudes, and NIHL in CBA/J mice. This protection was afforded, at least in part, through reduced cleavage of caspase 9 (CC9). Furthermore, MCU knockout mice on a hybrid genetic CD1 and C57/B6 background showed resistance to noise-induced seizures compared to wild-type littermates. Owing to the CD1 background, MCU knockouts and littermates suffer genetic high frequency hearing loss, but their IHCs remain intact. Noise-induced loss of IHC synaptic connections and reduction of auditory brainstem response (ABR) wave I amplitude were recovered in MCU knockout mice. These results suggest that cellular calcium influx during noise exposure leads to mitochondrial calcium overload *via* MCU and NCLX. Mitochondrial calcium overload, in turn, initiates cell death pathways and subsequent loss of hair cells and synaptic connections, resulting in NIHL.

## Introduction

Dysfunctional buffering of calcium ions in mitochondria or the cytosol is associated with pathological conditions (Williams et al., 2013). Specifically, dysregulation of cytosolic calcium homeostasis appears to contribute to noise-induced hearing loss (NIHL). This notion is supported by a noise-dependent elevation of calcium levels in sensory hair cells (Maurer et al., 1993; Fridberger et al., 1998; Oliver et al., 2001) and the fact that calcium channel blockers protect from NIHL (Maurer et al., 1993; Fridberger et al., 1998; Heinrich et al., 1999; Oliver et al., 2001; Minami et al., 2004; Shen et al., 2007; Zuo et al., 2008). An elevation of intracellular Ca^2+^ levels after noise exposure can be deduced from an increase in the Ca^2+^-binding protein calmodulin (CaM), a critical mediator of calcium signaling (Zuo et al., 2008). Such elevated calcium levels may contribute to sensory hair cell death, as noise exposure increases the phosphatase calcineurin (Minami et al., 2004) and triggers mitochondria-mediated cell death pathways (Vicente-Torres and Schacht, 2006).

Mitochondrial calcium has been postulated to regulate a wide range of processes involved in NIHL, including bioenergetics and cell death. The mitochondrial calcium uniporter (MCU) is an integral membrane protein located in the mitochondrial inner membrane. It is a major specific calcium channel for calcium uptake (Raffaello et al., 2012; Rizzuto et al., 2012). Excessive amounts of cellular calcium can rapidly enter the mitochondrial matrix through MCU (Raffaello et al., 2012; Rizzuto et al., 2012; Patron et al., 2013). MCU controls excitotoxicity (Qiu et al., 2013) and overexpression of MCU increases mitochondrial calcium uptake and sensitizes cells to apoptotic cell death (Patron et al., 2013). Excitotoxicity *via* an excess release of the neurotransmitter glutamate at inner hair cell (IHC) synapses has been linked to a loss of IHC connections to the auditory nerve (Puel et al., 1996). Glutamate overload results in a loss of function of type I afferent dendrites, and consequently the entry of Ca^2+^ triggers a cascade of metabolic events eventually leading to loss of function in type I spiral ganglion cells (SGCs). Furthermore, reduction of expression of the glutamate receptor AMPA reduces excitotoxicity in auditory neurons and correlates with auditory sensitivity (Chen et al., 2007, 2009). Conversely, extrusion of calcium from mitochondria is mediated primarily by a mitochondrial sodium calcium exchanger, encoded by the *NCLX* gene (Palty et al., 2012). Like MCU, NCLX is also localized in the mitochondrial inner membrane, where it regulates the mitochondrial calcium concentration and modulates intracellular calcium signaling. NCLX has been shown to be involved in neuronal death in a model of Parkinson disease (Gandhi et al., 2009; Palty et al., 2012).

**Table 1:** Experimental time line.

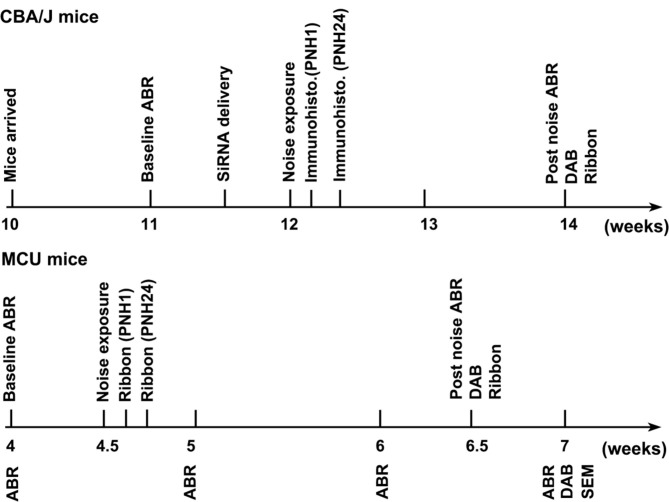

*CBA/J mice arrived at MUSC at the age of 10 weeks. Baseline ABRs were measured at the age of 11 weeks. At the age of 12 weeks, mice were exposed to noise. Two weeks after the noise exposure (14 weeks), mice were euthanized for surface preparations for hair cell counts (left ear) and ribbon counts (right ear) after post-noise ABR measurements. siRNA was delivered 72 h before the noise exposure. For immunohistochemistry, mice were euthanized 1 h or 24 h after the completion of noise exposure. MCU knockout mice and littermates were generated from breeders and ABRs were measured initially at the ages of 4, 5, 6, and 7 weeks to observe hearing function and some mice were euthanized for assessment of hair cell pathology using myosin-VIIa labeling and DAB staining as well as scanning electron microscopy. For noise-exposed mice, baseline ABRs were measured at the age of 4.5 weeks. Two weeks after the noise exposure (6.5 weeks), mice were euthanized for surface preparations of hair cell counts (left ear) and ribbon counts (right ear) after the post-noise ABR measurements. Some mice were euthanized 1 h or 24 h after the completion of noise exposure to evaluate ribbon synapses additional time points*.

While breakdown of calcium homeostasis appears to be crucial in the process leading to noise-induced sensory cell death and hearing loss, the role of mitochondrial calcium transporters and, particularly, the role of MCU and NCLX in the context of noise-induced hair cell death and hearing loss are unknown. We hypothesize that traumatic noise induces an increase in mitochondrial calcium *via* activation of MCU, thus stimulating calcium uptake, coupled with a reduction in calcium extrusion from mitochondria *via* depression of sodium calcium exchanger (NCLX) activity, which together result in mitochondrial calcium overload. This then triggers the initial mitochondrial dependent cell death pathways leading to hair cell death and hearing loss. To investigate this idea, we first examined the contribution of MCU in noise-induced loss of outer hair cells (OHCs) and synaptic ribbons and the subsequent effect on NIHL by inhibition of MCU using siRNA silencing techniques and the pharmacological inhibitor Ru306, a cell-permeable specific inhibitor of MCU that binds MCU with high affinity and blocks mitochondrial calcium influx in adult CBA/J mice. We then examined the expression of MCU and NCLX in noise-exposed cochlear tissue with focus on the OHCs. Furthermore, we employed MCU knockout mice to investigate IHC synapses. These studies are the first to explore the role of mitochondrial transporters in the pathogenesis of noise-induced hair cell loss, cochlear synaptopathy, and NIHL.

## Materials and Methods

### Animals

Male CBA/J mice at 10 weeks of age were purchased from The Jackson Laboratory. All mice had free access to water and a regular mouse diet (Irradiated Lab Diet #5V75) and were kept at 22 ± 1°C under a standard 12:12 h light-dark cycle to acclimate for 1 week before the experiments. MCU heterozygous mice on a hybrid CD1 and C57/B6 background were purchased from the Texas A&M Institute of Genomic Medicine (Pan et al., 2013; Murphy et al., 2014). MCU knockout and wild-type littermates were bred in the animal facility of the Children’s Research Institute (CRI) at the Medical University of South Carolina (MUSC). All mice were kept in the CRI animal facility at MUSC. All research protocols were approved by the Institutional Animal Care and Use Committee (IACUC) at MUSC. Animal care was under the supervision of the Division of Laboratory Animal Resources (DLAR) at MUSC. Table 1 illustrates general experimental time line.

### Noise Exposure

In this study, all CBA/J mice were exposed to 8–16 kHz at 101 dB sound pressure level (SPL) for 2 h and all MCU mice exposed to 2–8 kHz centered at 4 kHz for 2 h at 116 dB SPL unless otherwise stated. Unrestrained CBA/J male mice at 12 weeks of age (one mouse per stainless steel wire cage, approximately 9 cm^3^) were exposed to 101 dB SPL to induce permanent threshold shifts (PTSs) with losses of IHC ribbons and OHCs, but not IHCs, or to 108 dB SPL to induce severe PTS with losses of IHC ribbons, OHCs, and IHCs by 14 days after the noise exposure. MCU knockout mice and wild-type littermates were first exposed to OBN centered at 4 kHz for 2 h at 118 dB and 116 dB SPL. Since the exposure to 118 dB SPL induced death in MCU wild-type littermates in our previous experiments, we selected 116 dB SPL for the experiments using MCU knockouts and littermates. Due to hereditary high-frequency hearing loss in the CD1 strain, MCU knockouts and littermates were exposed at 4.5 weeks of age when hearing at 8 kHz remains intact. The sound exposure chamber was fitted with a loudspeaker (model 2450H; JBL) driven by a power amplifier (model XLS 202D; Crown Audio) fed from a CD player (model CD-200; Tascam TEAC American). Audio CD sound files were created and equalized with audio editing software (Audition 3; Adobe System Inc., San Jose, CA, USA). The background sound intensity of the environment surrounding the cages was 65 dB as measured with a sound level meter (model 1200; Quest Technologies). Sound levels for noise exposure are calibrated with a sound level meter at multiple locations within the sound chamber to ensure uniformity of the sound field and are measured before and after exposure to ensure stability. Control mice were kept in silence (without use of the loudspeaker) within the same chamber for 2 h.

### Auditory Brainstem Response and Measurement of ABR Wave I Amplitudes

Auditory brainstem responses (ABRs) were measured in anesthetized mice before and 2 weeks after noise exposure. Mice were anesthetized with an intra-peritoneal (IP) injection of a mixture of ketamine (100 mg/kg) and xylazine (10 mg/kg), and then placed in a sound-isolated and electrically shielded booth (Acoustic Systems, Austin, TX, USA). Body temperature was monitored and maintained near 37°C with a heating pad. Acoustic stimuli were delivered monaurally to a Beyer earphone attached to a customized plastic speculum inserted into the ear canal. Subdermal electrodes were inserted at the vertex of the skull (active), mastoid region under the left ear (reference), and mastoid region under the right ear (ground). ABRs were measured at 8, 16, and 32 kHz, as the system used is unable to test at lower frequencies such as 2 or 4 kHz. Tucker Davis Technology (TDT) System III hardware and SigGen/Biosig software (TDT, Alachua, FL, USA) were used to present the stimuli (15 ms duration tone bursts with 1 ms rise-fall time) and record the response. Upto 1,024 responses were averaged for each stimulus level. ABR wave I was used to determine ABR thresholds for each frequency. Thresholds were determined for each frequency by reducing the intensity in 10-dB increments and then in 5-dB steps near threshold until no organized responses were detected. Thresholds were estimated between the lowest stimulus level where a response was observed and the highest level without response. All ABR measurements were conducted by the same experimenter. The ABR thresholds were assigned by an expert who was blinded to the treatment conditions. The waveforms were saved and analyzed *post hoc* to measure the wave I amplitudes.

### Drug Administration *via* Intra-Peritoneal Route to Mice

Ru360 (Calbiochem, 557440) was dissolved in 0.9% oxygenated saline (10 mg/mL Ru360) as a stock solution, aliquoted, and stored at −20°C. The stock solution was diluted with 0.9% oxygenated saline immediately before being injected into animals. Two concentrations of Ru360 (120 μg/kg and 240 μg/kg) were tested in our preliminary study, based on the literature (Sanganahalli et al., 2013). The final selected concentration of R360 was 240 μg/kg for use in this study. The mice used for the experiments designed to observe the evolution of ABR threshold shifts and hair cell counts were given five IP injections of Ru360 30 min before the noise exposure and 30 min, 4 h, 24 h, and 30 h after the end of noise exposure. Control animals received the same volume of 0.9% oxygenated saline on the same schedule. The mice used for immunohistochemistry to determine protein expression received only two IP injections of Ru360 at 30 min before and 30 min after the noise exposure.

### Intra-Tympanic Delivery of MCU siRNA or MitoTracker *in vivo*

MCU siRNA (siMCU; Thermo Fisher, 103464) or siControl (Thermo Fisher, 4390843) was delivered locally *via* intra-tympanic application as previously described (Chen et al., 2013; Oishi et al., 2013). Briefly, after anesthesia, a retroauricular incision was made to approach the temporal bone. The otic bulla was identified ventral to the facial nerve and a shallow hole was made in the thin part of the otic bulla with a 30-G needle and enlarged with a dental drill to a diameter of 2 mm in order to visualize the round window. A customized sterile micro medical tube was inserted into the hole just above the round window niche (RWN) to slowly deliver 10 μL (0.6 μg) of pre-designed siRNA or MitoTracker (0.5 μM, Invitrogen, M7512) to completely fill the mouse RWN. After the siRNA or MitoTracker was delivered, the hole was covered with the surrounding muscle. Finally, the skin incision was closed with tissue adhesive. The animal was allowed to rest in the surgical position for an additional hour after the procedure. Seventy-two hours after siRNA delivery, the animals were exposed to noise for 2 h. Based on our previous experiments, local intra-tympanic delivery of siRNA results in temporary elevation of thresholds that completely recovers to baseline by 72 h (Oishi et al., 2013; Zheng et al., 2014; Yuan et al., 2015). Therefore, noise exposure was performed at least 72 h after siRNA delivery. Forty-eight hours after MitoTracker delivery, control mice without noise exposure were euthanized for immunoassays to assess co-localization of MitoTracker with NCLX on surface preparations.

### Surface Preparations and Myosin VIIa-DAB Staining of Cochlear Epithelia for Hair Cell Counts

This procedure was described in our previous reports (Chen F.-Q. et al., 2012; Zheng et al., 2014; Yuan et al., 2015). Briefly, 2 weeks after the end of noise exposure and after the final ABR measurement, the temporal bones were removed immediately following euthanasia and locally perfused gently with a solution of 4% paraformaldehyde in phosphate buffered saline, pH 7.4 (PBS) after removing the stapes and opening the oval and round windows and kept in this fixative overnight at 4°C. The cochleae were then rinsed in PBS. Before decalcification in a 4% solution of sodium EDTA (adjusted with HCl to pH 7.4), the apical and middle turns of the otic capsule were removed from each cochlea. The EDTA solution was changed daily for 3 days and maintained at 4°C. Following decalcification, the cochleae were placed in 3% hydrogen peroxide for 2.5 h to quench endogenous peroxidases. After incubation in a solution for blocking non-specific antibody binding overnight at 4°C, the tissues were incubated with a primary antibody (rabbit polyclonal anti-myosin VII, Proteus Bioscience, 25-6790) at a 1:100 dilution for 4 days at 4°C on a Nutator mixer, washed in PBS, and then incubated overnight at 4°C with secondary antibody (biotinylated goat anti-rabbit) at a 1:100 dilution. The specimens were rinsed again and then incubated in ABC solution (Vector Laboratories, PK-4001) overnight. Following another washing, the cochleae were incubated in DAB for 3 h, as necessary for sufficient staining intensity, followed by washing to stop the DAB reaction. Finally, the cochleae were micro-dissected under a microscope into apical, middle, and basal segments and mounted on slides with Fluoromount-G mounting medium. Images were taken with a Zeiss AxioCam MRc5 camera with Axioplan 2 imaging software with a Zeiss microscope for hair cell counts. Unless otherwise specified, all chemicals and reagents used were purchased from Sigma-Aldrich.

Mapping of frequencies as a function of distance along the length of the cochlear spiral was done using the ImageJ plugin. In addition, we also calculated with equation [d = 156.5–82.5 × log (f)] from Müller’s article (Müller et al., 2005). They are in agreement with literature (Viberg and Canlon, 2004).

### Hair Cell Counts on Cochlear Epithelia From the Adult Mouse

Hair cells were counted from captured images using the 40×-magnification lens on the Zeiss microscope from the apex through the base of the DAB-stained surface preparations. The lengths of the cochlear epithelia were measured and recorded in millimeters. Both outer and IHCs were counted from the apex to the base of the mouse cochlear epithelium. Percentages of hair cell loss in each 0.5-mm length of epithelium were plotted as a function of the cochlear length as a cytocochleogram (Chen F.-Q. et al., 2012; Zheng et al., 2014).

### Immunocytochemistry for Cochlear Paraffin Sections

Following decalcification with 4% EDTA, each cochlea was transferred to 70% ethanol and embedded in paraffin for sectioning. Five-micrometer sections were routinely deparaffinized in xylene and rehydrated in alcohol. The sections were incubated with target retrieval solution (Dako, S2367) in a steamer (Oster, CKSTSTMD5-W) for 10 min and then 3% hydrogen peroxide solution for 10 min and protein block solution (Dako, 0909) for 20 min at room temperature. All primary antibodies were first optimized by titration with five different concentrations at two pH values (pH 6 and 9). Then primary antibodies (MCU at 1:200; Sigma-Aldrich, HPA016480) were applied and incubated overnight in a humid chamber at 4°C, followed by incubation with a biotinylated secondary antibody (Vector Laboratories, Torrance, CA, USA) for 30 min and ABC reagent (Vector Laboratories, Torrance, CA, USA) for 30 min. Immunocomplexes of horseradish peroxidase were visualized by DAB reaction, and sections were counterstained with hematoxylin before mounting.

### Scanning Electron Microscope

Temporal bones of MCU mice at the age of 7 weeks were removed after cardio-vascular perfusion of anesthetized mice with a mixture of 4% paraformaldehyde and 2% glutaraldehyde in 0.1 M cold phosphate buffer, pH 7.4. The temporal bones were then locally perfused gently with the same fixative after removing the stapes and opening the oval and round windows and were kept in this fixative overnight at 4°C. The samples were washed with the phosphate buffer and decalcified with 4% EDTA, pH 7.4, for 72 h. After decalcification, each cochlea was dissected by removing the softened otic capsule, stria vascularis, Reissner’s membrane, and tectorial membrane. The remaining tissues, including the modiolus and cochlear sensory epithelium were post fixed with 1% osmium tetroxide-1.5% ferrocyanide for 2 h in the dark, then dehydrated in increasing concentrations of ethanol from 70% to 100%, and dried with Hexamethyldisilazane until it evaporates. The specimens were micro-dissected by removing the modiolus and divided into three segments (apex, middle, and base). Each specimen was mounted on a scanning electron microscopy stub and sputter coated with 10 nm gold alloy. Cochlear epithelia were viewed and photographed with a JEOL 1510 scanning electron microscope (SEM).

### Immunocytochemistry on Cochlear Surface Preparations

We have followed a procedure as previously described (Chen F.-Q. et al., 2012; Zheng et al., 2014; Yuan et al., 2015). Briefly, depending on the time points, mice were euthanized either 1 h or 24 h after completion of the exposure. The temporal bones were removed immediately following euthanasia, perfused locally with a fresh solution of 4% paraformaldehyde in PBS, pH 7.4, and kept in this fixative overnight at 4°C. The cochleae were then rinsed in PBS prior to decalcification with 4% EDTA. Following 72 h decalcification, each cochlea was dissected by removing the softened otic capsule, stria vascularis, Reissner’s membrane, and tectorial membrane. The remaining tissue, including the modiolus and cochlear sensory epithelium, was permeabilized in fresh 3% Triton X-100 solution for 30 min at room temperature. The specimens were washed three times (10 min each) with PBS and blocked with 10% normal goat serum for 30 min at room temperature. The tissues were incubated at 4°C for 48 h with the following primary antibodies: MCU (1:50; Sigma-Aldrich, HPA016480), NCLX (1:50, Sigma-Aldrich, HPA040668), and cleaved caspase 9 (CC9; 1:50, Cell Signaling Technology, 9509). After three washings, the tissues were incubated with the Alexa-Fluor-594- or Alexa-Fluor-488-conjugated secondary antibody at a concentration of 1:200 at 4°C overnight in darkness. The specimens were then washed three times with PBS and incubated with Alexa Fluor 488 phalloidin at a concentration of 1:100 for 1 h in darkness at room temperature. After at least three final washes with PBS, the specimens were micro-dissected in PBS by removing the modiolus and divided into three segments (apex, middle, and base). Each segment was mounted on slides with Fluoro-gel with Tris buffer (Electron Microscopy Sciences, 17985-10). Control incubations were routinely processed without primary antibody treatments. Immunolabeled images were taken using a Zeiss laser confocal microscope (Zeiss LSM 880) or Leica SP5 confocal microscope.

### Immunocytochemistry for Synaptic Ribbons on Cochlear Surface Preparations

Depending on the time points, CBA/J mice were euthanized at either 1 h or 14 days and MCU knockouts and littermates were euthanized at 1 h, 24 h, and 14 days, after completion of the exposure. The temporal bones were removed immediately following euthanasia, perfused locally with a fresh solution of 4% paraformaldehyde in PBS, pH 7.4 and fixed for 1 h at room temperature. After decalcification with 4% EDTA for 3 days, each cochlea was dissected by removing the softened otic capsule, stria vascularis, Reissner’s membrane, and tectorial membrane. The remaining tissue, including the modiolus and cochlear sensory epithelium, was permeabilized in fresh 3% Triton X-100 solution for 30 min at room temperature. The specimens were washed three times (10 min each wash) with PBS and blocked with 10% normal goat serum for 30 min at room temperature and then incubated in darkness at 37°C overnight with primary monoclonal mouse anti-CtBP2 IgG1 at 1:200 (BD Biosciences, 612044) and monoclonal mouse anti-GluA2 IgG2a at 1:2,000 (Millipore, MAB397). After washing three times, the tissues were incubated with the Alexa-Fluor-594- and Alexa-Fluor-488-conjugated secondary antibody at a concentration of 1:1,000 at 37°C for 1 h in darkness. After washing three times, the tissues were re-incubated with Alexa-Fluor-conjugated secondary antibodies for an additional 1 h at 37°C to increase the immunolabeling for CtBP2 (Wan et al., 2014; Hill et al., 2016). Following three washings, the tissues were incubated in darkness at 4°C overnight with polyclonal rabbit anti-myosin VIIa at 1:200 (Proteus Biosciences, 25-6790). Then following washing steps, the tissues were incubated with Alexa Fluor 350-conjugated secondary antibody at a concentration of 1:200 at 4°C overnight in darkness. For all immunolabeling samples, after at least three final washes with PBS, the specimens were micro-dissected in PBS by removing the modiolus and divided into three segments (apex, middle, and base). Each segment was mounted on slides with Fluoro-gel with Tris buffer (Electron Microscopy Sciences, 17985-10). Immunolabeled images were taken with a 63×-magnification lens under identical Z-stack conditions using a Zeiss LSM 880 confocal microscope.

### Quantification of the Immunofluorescence Signals From Outer Hair Cells of Surface Preparations

Immunohistochemistry is well accepted as a semi-quantitative methodology when used with careful consideration of the utility and semi-quantitative nature of these assays (Taylor and Levenson, 2006; Walker, 2006). The specificity of antibodies must be first detected by Western blot analysis. An antibody showing only a single band with the correct molecular weight was then used for immunolabeling on surface preparations and quantification of signaling in OHCs. The regions of interest were outlined within individual OHCs based on the counterstaining. The grayscale value was determined in only the hair cells to quantify the change in fluorescence intensity. This procedure provided quantitative measurements that are not confounded by protein expression in other cell types of the cochlea.

Immunolabeling for MCU, NCLX, and CC9 on surface preparations was quantified from original confocal images with 8-bit grayscale values, each taken with a 63×-magnification lens under identical conditions and equal parameter settings for laser gains and photomultiplier tube (PMT) gains within linear ranges of the fluorescence, using ImageJ software (National Institutes of Health, USA). The cochleae from the different groups were fixed and stained simultaneously with identical solutions and processed in parallel. All surface preparations were counterstained with Alexa Fluor 488 phalloidin for labeling OHC structure in order to identify the comparable parts of the OHCs in confocal images. The regions of interest of individual OHC cell bodies were outlined with the circle tool based on the phalloidin staining. The immunolabeling of MCU, NCLX, and CC9 in OHCs was measured in the upper-basal region of surface preparations, corresponding to sensitivity to 22–32 kHz, in 0.12-mm segments, each containing about 60 OHCs. The intensity of the background fluorescence was subtracted and the average fluorescence per cell was then calculated. For each repetition, the relative grayscale values were determined by normalizing the ratio to control.

### Quantification of the Immunolabeled Ribbons From Z Projections on Surface Preparations

We have followed a procedure as previously described (Hill et al., 2016). Immunofluorescence of CtBP2 on surface preparations was quantified from original confocal images, each taken with a 63×-magnification lens under identical Z-stack conditions in 0.25-mm intervals and equal parameter settings for laser gains and PMT gains. The z-stack images in each 0.12-mm segment (containing about 16 IHCs) were captured from cochlear surface preparations. The number of synaptic ribbon particles was counted using ImageJ software (National Institutes of Health, USA). Briefly, the background of the images was subtracted, the noise was despeckled once, and the threshold was set to isolate the immunolabeling of ribbon signals. The image was then converted to a binary file and the number of ribbon particles was counted using the 3D Object Counter and divided by the total number of IHC nuclei within the image. The number of functional synapses, identified by juxtaposed CtBP2 and GluA2, were manually counted by visualizing the presence of CtBP2 co-localization with GluA2.

### Extraction of Total Cochlear Protein and Liver Protein

Cochlear or liver tissue was rapidly removed and dissected in ice-cold PBS containing complete™ mini EDTA-free protease inhibitor cocktail tablets (Roche Diagnostic, 11836170001) at pH 7.4. To extract total protein, tissue from two cochleae from one mouse or a small piece of liver was homogenized in ice-cold radioimmunoprecipitation assay (RIPA) lysis buffer (Sigma-Aldrich, R0278) plus Phosphatase Inhibitor Cocktails II and III, and Roche Protease Inhibitor (cocktail tablets) by using a glass/glass micro Tissue Grind pestle and vessel for 30 s. After 30 min on ice, tissue debris was removed by centrifugation at 15,000× *g* at 4°C for 10 min and the supernatants were retained as the total protein fractions. Protein concentrations were determined using the Bio-Rad Protein Assay dye reagent with bovine serum albumin as a protein standard.

### Extraction of Protein From Formalin-Fixed Sensory Epithelia

We followed a procedure as previously described (Hill et al., 2016). Cochleae were rapidly removed and perfused with 4% paraformaldehyde and incubated for 2 h at room temperature (25°C). The cochleae were then rinsed in PBS and decalcified in a 4% solution of sodium EDTA for 3 days at 4°C, with the EDTA solution changed daily. Following decalcification, the micro-dissected sensory epithelia from three mice were placed in 1.5-mL collection tubes with 100 μL of extraction buffer EXB plus (Qproteome FFPE Tissue kit Qiagen, 37623) supplemented with β-mercaptoethanol. Glass micro grinder pestles were used to grind the tissue for 3 min. The tubes were sealed with a sealing clip and vortexed. The samples were incubated on ice for 5 min, followed by repeat vortexing. The tubes were then incubated for 20 min at 100°C on a heating block. After this incubation, the tubes were incubated for 2 h at 80°C with agitation at 750 rpm (Eppendorf) and then allowed to cool at 4°C for 1 min. Finally, the samples were centrifuged at 14,000× *g* at 4°C for 15 min. The supernatant containing the extracted proteins was transferred to a new tube. Protein concentrations were determined using the Bio-Rad RC DC protein assay (Invitrogen, 500-0119) with bovine serum albumin as a protein standard.

### Cell Culture

HEI-OC1, an inner ear cell line, was provided by Dr. Kalinec, from UCLA, Los Angeles, CA, USA. The cell line was cultured on plastic culture dishes under permissive conditions (33°C, 10% CO_2_) in high-glucose Dulbecco’s Modified Eagle’s Medium (DMEM; Gibco BRL, Gaithersburg, MD, USA) containing 10% fetal bovine serum (FBS; Gibco BRL) and 100 U/mL penicillin to proliferate.

### Protein Extraction From Cultured Cells

HEI-OC1 cells were taken out of the incubator and treated with trypsin-EDTA (Thermo Fisher Scientific, 25200056) for 5 min. The trypsin was diluted with 10 mL of DMEM. The collected cells were transferred to a 15-mL conical tube (Corning, 430052) and centrifuged at 1,000× *g* for 5 min. After the medium was removed, the pellets of cells with 500 μL medium were transferred to 1.5-mL Eppendorf tubes (Thermo Fisher Scientific, 05408133). The cells were washed with 1 mL of PBS (Invitrogen, 20012) and centrifuged again at 1,000× *g* for 5 min. After removing the PBS, 100 μL of RIPA buffer was added to the cell pellets and the tubes were vortexed for 5 s and incubated for 20 min on ice. The RIPA buffer contained 860 μL RIPA buffer base (Sigma-Aldrich, R0278), 100 μL Protein Inhibitor Cocktail (Roche, 11836170001), 5 μL PMSF (Sigma-Aldrich, P7626), 10 μL Phosphatase Inhibitor Cocktail 2 (Sigma-Aldrich, P5726), 10 μL Phosphatase Inhibitor Cocktail 3 (Sigma, P0044). The supernatants were collected in a clean, labeled tube and kept at −80°C after centrifugation.

### Western Blot Analysis

Protein samples (30 μg) were separated by SDS-PAGE. After electrophoresis, the proteins were transferred onto a nitrocellulose membrane (Pierce, USA) and blocked with 5% solution of nonfat dry milk in PBS-0.1% Tween 20 (PBS-T). The membranes were incubated with anti-MCU (1:1,000) or anti-NCLX (1:1,000) at 4°C overnight and then washed three times (10 min each) with PBS-T buffer. Membranes were incubated with an appropriate secondary antibody at a concentration of 1:2,500 for 1 h at room temperature. Following extensive washing of the membrane, the immunoblots were visualized by SuperSignal^®^ West Dura Extended Duration Substrate or Pierce^®^ ECL Western Blotting Substrate (Thermo Fisher Scientific, Waltham, MA, USA). The membranes were then stripped and relabeled for GAPDH (1:10,000; Millipore, MAB374) at a concentration of 1:20,000 as a control for sample loading.

X-ray films of Western blots were scanned and analyzed using ImageJ software. The band densities were first normalized to the background. Next, the probing protein/GAPDH ratio was calculated from the band densities run on the same gel. Finally, the difference in the ratio of the control and experimental bands was tested for statistical significance.

### Statistical Analysis

Data were analyzed using IBM SPSS Statistics Premium V21 and GraphPad software (GraphPad Software Inc.,) for Windows. Biological sample sizes were determined based on the variability of measurements and the magnitude of the differences between groups, as well as experience from our previous studies, with stringent measurements of difference. Data of OHC loss along the length of the cochlear spiral were analyzed with repeated measures one-way analysis of variance (ANOVA) with Tukey’s multiple comparisons using IBM SPSS Statistics. The rest analysis was done using GraphPad. Data with multiple comparisons were evaluated by one-way ANOVA with multiple comparisons. Differences for single-pair comparisons were analyzed using two-tailed unpaired Student’s *t*-tests. Data for relative ratios of single-pair comparisons were analyzed with one-sample *t*-tests. The difference of death rate of MCU mice vs. wild-type littermates was analyzed using Chi-square test (and Fisher’s exact test). A *p*-value < 0.05 was considered statistically significant. Data are presented as means ± SD or SEM based on the sample size and variability within groups. Sample sizes are indicated for each figure.

## Results

### Inhibition of Mitochondrial Calcium Uniporter in CBA/J Mice by Pretreatment With siRNA or Ru360 Protects Against Noise-Induced Outer Hair Cell Loss and Permanent Hearing Loss

**Figure 1:**
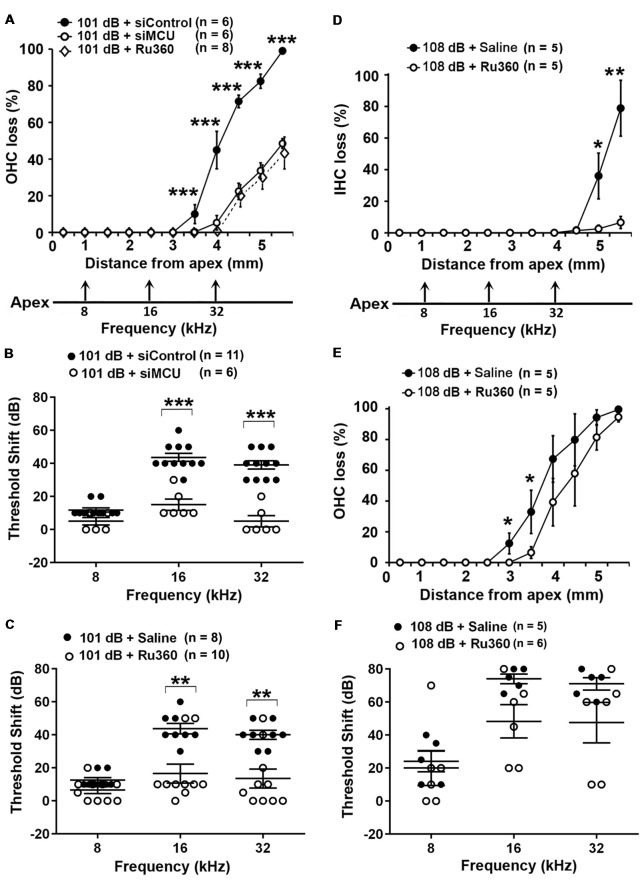
Inhibition of mitochondrial calcium uniporter (MCU) *via* siRNA silencing or the pharmacological inhibitor Ru360 attenuated noise-induced hair cell loss and hearing loss. **(A)** Noise-induced outer hair cell (OHC) loss was reduced by siMCU pretreatment as well as by Ru360 treatment. The distance along the cochlear duct correlating with the frequencies of 8, 16, and 32 kHz is indicated. Data are presented as means ± SD; ***p < 0.001. **(B)** Pretreatment with siMCU attenuated 101-dB-noise-induced auditory threshold shifts measured 14 days after the exposure. Data are presented as individual points and means ± SD, ***p < 0.001. **(C)** Treatment with Ru360 also attenuated 101-dB-noise-induced auditory threshold shifts measured 14 days after the exposure. Data are presented as individual points and means ± SD, **p < 0.01. **(D)** Treatment with Ru360 attenuated 108-dB-noise-induced inner hair cell (IHC) loss. The distance along the cochlear duct correlating with the frequencies of 8, 16, and 32 kHz is indicated. Data are presented as means ± SD, *p < 0.05, **p < 0.01. **(E)** Treatment with Ru360 attenuated 108-dB-noise-induced OHC loss at 3 and 3.5 mm from the apex. Data are presented as means ± SD, *p < 0.05. **(F)** Treatment with Ru360 did not attenuate 108-dB-noise-induced auditory threshold shifts measured 14 days after the exposure. Data are presented as individual points and means ± SD, *n* in all figures indicates the number of mice per group; left cochlea was assessed per mouse.

Based on our hypothesis, we first tested if inhibition of MCU could attenuate NIHL. Exposure of mice to the octave band noise (OBN; 8–16 kHz) induces loss of sensory hair cells following a base-to-apex gradient with losses beginning at the basal turn. Such a pattern of damage is similar to that seen in mice exposed to broadband noise (2–20 kHz) when examined 14 days after the completion of noise exposure (Figure 1A; Yuan et al., 2015; Hill et al., 2016). Using our lab’s established technique for intra-tympanic delivery of siRNA into adult mouse cochleae (Oishi et al., 2013), we first determined the appropriate concentration of siMCU to be used in this study. The 0.6-μg concentration of siMCU was selected from preliminary experiments based on the assessment of the efficacy from two concentrations (0.3 and 0.6 μg). Immunolabeling for MCU on cochlear surface preparations revealed 25% reduction of MCU in OHCs 72 h after 0.6-μg siRNA delivery compared to untreated controls (Supplementary Figures S1A,B). Additionally, Western blots with formalin-fixed sensory epithelium from pooled tissues also showed a significant 30% reduction in MCU band densities 72 h after siRNA delivery; Supplementary Figure S1C). Next, we found that pretreatment with siMCU reduced noise-induced OHC loss by 50% at 3.5–5.5 mm from the apex at 14 days after the exposure (Figure 1A). Noise-induced auditory threshold shifts were also significantly attenuated both at 16 and 32 kHz in the siMCU-pretreated group (Figure 1B).

Finally, we inhibited MCU with the specific inhibitor Ru360. Based on the literature, the doses of Ru360 used *in vivo* were tested at two concentrations (120 μg/kg and 240 μg/kg) in a preliminary study (Sanganahalli et al., 2013). CBA/J mice at 12 weeks of age tolerated Ru360 at either dose for five IP injections over 2 days without loss of body weight or changes in fur appearance. Ru360 also did not alter baseline hearing thresholds. However, treatment with Ru360 at 120 μg/kg did not significantly attenuate noise-induced auditory threshold shifts. We therefore chose the 240-μg/kg dose of Ru360 for assessment of a protective effect against NIHL. After treatment with Ru360 at 240 μg/kg, noise-induced auditory threshold shifts at both 16 and 32 kHz were significantly reduced (Figure 1C). Furthermore, treatment with Ru360 also reduced the extent of OHC loss by 50% at 3.5–5.5 mm from the apex 2 weeks after the noise exposure (Figure 1A). Additionally, we tested Ru360 treatment against a more severe noise damage paradigm (108 dB SPL for 2 h) that induced IHC loss at the basal turn at 4.5–5.5 mm from the apex (Figure 1D). Treatment with Ru360 almost completely blocked IHC loss (Figure 1D). Meanwhile, treatment with Ru360 also reduced OHC loss from the 108-dB exposure from 3–3.5 mm (*p* = 0.053), but not from 4–5.5 mm from the apex (Figure 1E). However, 108-dB-SPL-induced auditory threshold shifts were not significantly attenuated at 8, 16, or 32 kHz (Figure 1F). These results pointed out that blockade of MCU function can prevent moderate noise-induced permanent hearing loss, but not severe noise-induced permanent hearing loss.

### Inhibition of Mitochondrial Calcium Uniporter in CBA/J Mice by Treatment With Ru360 or siRNA Reduces the Noise-Induced Loss of IHC Ribbons and ABR Peak I Amplitudes

**Figure 2:**
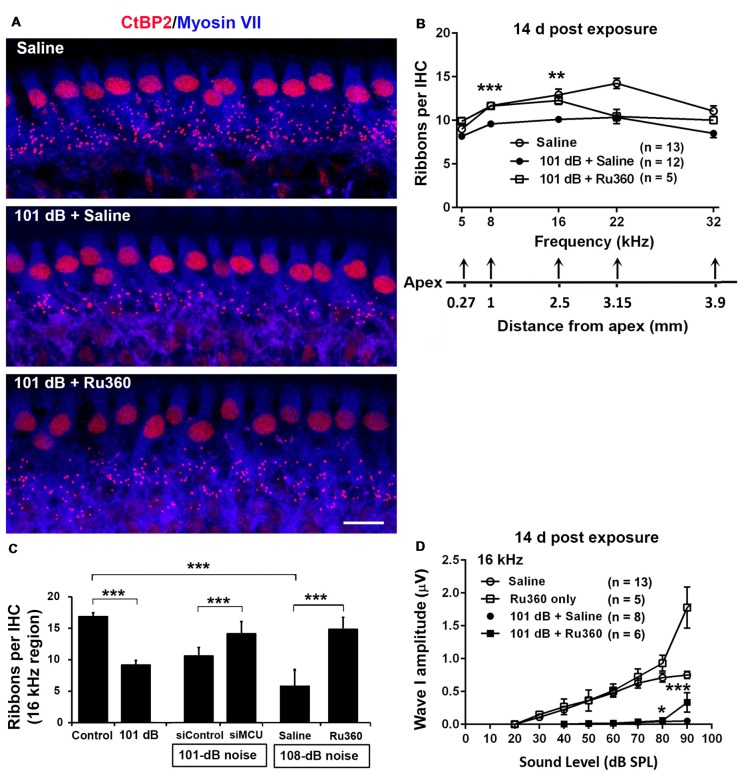
Inhibition of MCU *via* Ru360 or siMCU attenuates noise-induced loss of synaptic ribbons and wave I amplitudes after the completion of noise exposure. **(A)** Representative images revealed immunolabeling for CtBP2 examined 14 days after noise exposure. Images are comprised of 40 Z-stack projections taken from the middle turn corresponding to sensitivity to 16 kHz. Blue: myosin-VIIa labeled IHCs, red: CtBP2-labeled synaptic ribbons and nuclei of IHCs; scale bar = 10 μm. **(B)** Quantification of CtBP2-immunolabeled ribbon particles in IHCs corresponding to 5, 8, 16, 22, and 32 kHz showed significant reduction examined 14 days after noise exposure at all frequencies except 5 kHz (see Supplementary Table S1 for detailed statistical values). Treatment with Ru360 prevented noise-induced synaptic ribbon loss at 8 and 16 kHz. The distance along the cochlear duct correlating with the frequency regions is indicated. Data are presented as means + SEM. **p < 0.01, ***p < 0.001 for 101 dB + Saline vs. 101 dB + Ru360. **(C)** CtBP2-immunolabeled ribbon particles in IHCs at 16 kHz region also decrease examined 1 h after noise exposure that partially prevented with siMCU pretreatment; *n* = 4 mice per group with one cochlea used per mouse. Treatment with Ru360 also attenuated higher intensity noise sound pressure level (108-dB-SPL)-induced synaptic ribbon loss; *n* = 6 mice per group with one cochlea used per mouse. Data are presented as means + SD. ***p < 0.001. **(D)** Ru360 treatment alone increased wave I amplitudes at sound intensities of 90 dB SPL. Noise-reduced wave I amplitudes at sound intensities of 80 and 90 dB SPL were rescued by treatment with Ru360. Data are presented as means + SEM, *p < 0.05, ***p < 0.001 corresponds to 101 dB + Saline vs. 101 dB + Ru360. In panels **(B,D)** *n* indicates the number of mice per group; the left cochlea was used from each mouse for these experiments.

To determine if blockade of MCU could attenuate noise-induced loss of IHC synaptic ribbons, ribbon numbers were counted and peak I amplitudes were measured 14 days after the completion of noise exposures. Based on our previous characterization of noise-induced loss of IHC pre-synaptic ribbons and ribbon synapses in CBA/J mice, we found significant reduction of ribbons juxtaposed with presynaptic ribbons (labeled with CtBP2) and glutamate receptors (labeled with GluA2) when examined 14 days after either noise exposure condition (101 dB SPL or 108 dB SPL; Hill et al., 2016). In this study, we focused on presynaptic ribbons (labeled with CtBP2). Noise exposure decreased synaptic ribbons at areas corresponding to 8–32 kHz compared to age-matched unexposed controls (Saline). One-way ANOVA analysis of three groups (Control, 101-dB noise + Saline, and 101-dB noise + Ru360) showed a significant difference at 8 kHz, 16 kHz, 22 kHz, and 32 kHz, but not at 5 kHz (Figures 2A,B, detailed statistical values see Supplementary Table S1), while treatment with Ru360 significantly protected ribbons from damage by noise exposure at 8 kHz (*p* = 0.0001) and 16 kHz (*p* = 0.003), not at 5, 22, or 32 kHz (Figures 2A,B). Such protection of IHC synaptic ribbons matched age-matched mice without noise exposure particularly at lower frequencies (Figure 2B). Additionally, we also assessed CtBP2-labeled synaptic ribbons in the region of 16 kHz by treatment with siMCU or Ru360 examined 1 h after the completion of 101-dB or 108-dB noise exposure. The 101-dB exposure induced about 50% reduction, from 18 ribbons per IHC to 9, while pretreatment with siMCU reduced the loss of ribbons, bringing the average ribbon count up to 14 ribbons per IHC (Supplementary Figures S2A,B). The 108-dB exposure induced 66% reduction of ribbons, from 18 ribbons to 5 ribbons per IHC, whereas the treatment with Ru360 attenuated ribbon loss, restoring the ribbon number to 14 per IHC (Figure 2C). Furthermore, we assessed wave I amplitudes, which reflect the summed activity of auditory nerve fibers. Since loss of OHCs is a confounding factor affecting wave I amplitudes, we only measured at 16 kHz, which is one of the most sensitive frequencies of the auditory spectrum in mice, corresponding to a region where no OHC loss was found 14 days after the exposure. Noise exposure significantly diminished ABR wave I amplitudes from 30 dB to 100 dB SPL compared to age-matched mice without noise exposure (Saline control; detailed statistical values see Supplementary Table S2; Control vs. 101 dB noise). Treatment with Ru360 alone also elevated ABR wave I amplitudes at 70–100 dB SPL (detailed statistical values see Supplementary Table S2; Control vs. Ru360 only). Inhibition of MCU by treatment with Ru360 significantly reversed the noise-induced decrease in peak I amplitudes at sound intensities of 80, 90, and 100 dB SPL (Figure 2D, for detailed statistical values see Supplementary Table S2; 101 dB + Saline vs. 101 dB + Ru360).

### Noise Trauma Increases Mitochondrial Calcium Uniporter in the Basal Turn of Outer Hair Cells of CBA/J Mice

**Figure 3:**
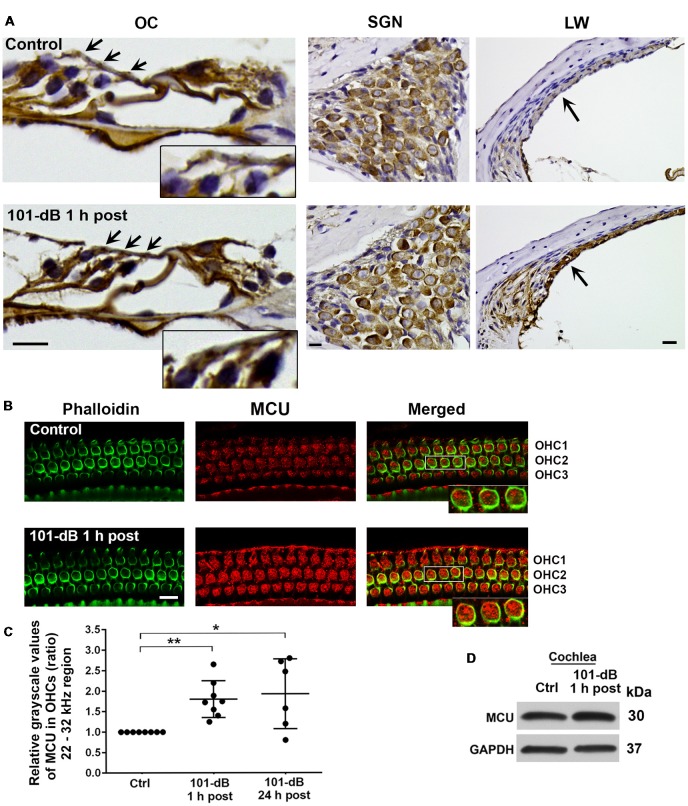
Noise exposure increased immunolabeling for MCU in OHCs and the stria vascularis of the basal turn. **(A)** Paraffin sections of the adult CBA/J mouse inner ear revealed an increase in immunolabeling for MCU in DAB-stained OHCs (arrows and enlarged image inserts) in the organ of Corti (OC) and the stria vascularis (arrow) in the lateral wall, and no obvious change in spiral ganglion neurons (SGNs) examined 1 h after completion of the 101-dB noise exposure. These images were taken with 40×-magnification lens and are representative of five individual mice per group; scale bar = 10 μm. **(B)** Representative images for MCU in OHCs of surface preparations stained with phalloidin when processed 1 h after completion of the noise exposure. An enlarged image of three OHCs better illustrates the immunolabeling for MCU. Images were taken from the area of the basal turn corresponding to 22–32 kHz; OHC1, 2, 3 indicate the three rows of OHCs, scale bar = 10 μm. **(C)** Quantification of immunolabeling for MCU in OHCs in the 22–32 kHz region showed a significant increase when processed 1 h after and 24 h after completion of the exposure. Data are presented as individual points and means ± SD; *p < 0.05, **p < 0.001. Control: *n* = 8, 101-dB 1 h post: *n* = 8, 101-dB 24 h post: *n* = 6 with one cochlea from each mouse in the group. **(D)** Immunoblots using total cochlear homogenates of CBA/J mice revealed no difference in MCU band densities between control (Ctrl) and noise-exposed mice processed 1 h after completion of the noise exposure (101-dB 1 h post). GAPDH was used as a loading control; *n* = 8 mice per group.

Since inhibition of MCU activity by siRNA treatment and pharmacological inhibitor Ru360 attenuated noise-induced loss of ribbons and hair cells as well as NIHL, we further assessed the expression and localization of MCU in cochlear paraffin sections of CBA/J mice processed 1 h after completion of a noise exposure (OBN, 101 dB SPL). Immunohistochemistry revealed increased MCU labeling in OHCs of the organ of Corti (OC, insert enlarged images) and in the stria vascularis, but no obvious changes in spiral ganglion neurons (SGNs; Figure 3A). In order to quantify the immunolabeling for MCU in OHCs, we conducted immunohistochemistry on cochlear surface preparations. MCU immunolabeling was stronger in OHCs of the basal turn when processed 1 h after noise exposure and was sustained until at least 24 h after the exposure. Quantification from original confocal images of the area of the basal turn corresponding to 22–32 kHz revealed that MCU in OHCs increased by 80% when examined at 1 h and 24 h after the exposure compared to age-matched controls (Figures 3B,C); although there was a larger variation at 24 h, there was no significant difference between the 1-h and 24-h time points. Furthermore, since the specificity of the MCU antibody from Sigma-Aldrich had only been confirmed in human tissue, we tested its applicability to mouse tissue by Western blots first using homogenates from HEI-OC1 cells, which showed a single band with a molecular weight at 30 kDa (Supplementary Figure S2A). We then used liver homogenates from MCU knockouts and wild-type littermates. A specific band for MCU was detected at 30 kDa in samples from MCU wild-type liver tissues, but not in MCU knockout mice (Supplementary Figure S2B). Additionally, immunoblots using total cochlear homogenates from CBA/J mice revealed no difference between the MCU band density of mice with and without noise exposure (Figure 3D).

### Noise Trauma Depresses Mitochondrial Sodium Calcium Exchanger in the Basal Turn of Outer Hair Cells in a Time-Dependent and Noise-Intensity-Dependent Manner

**Figure 4:**
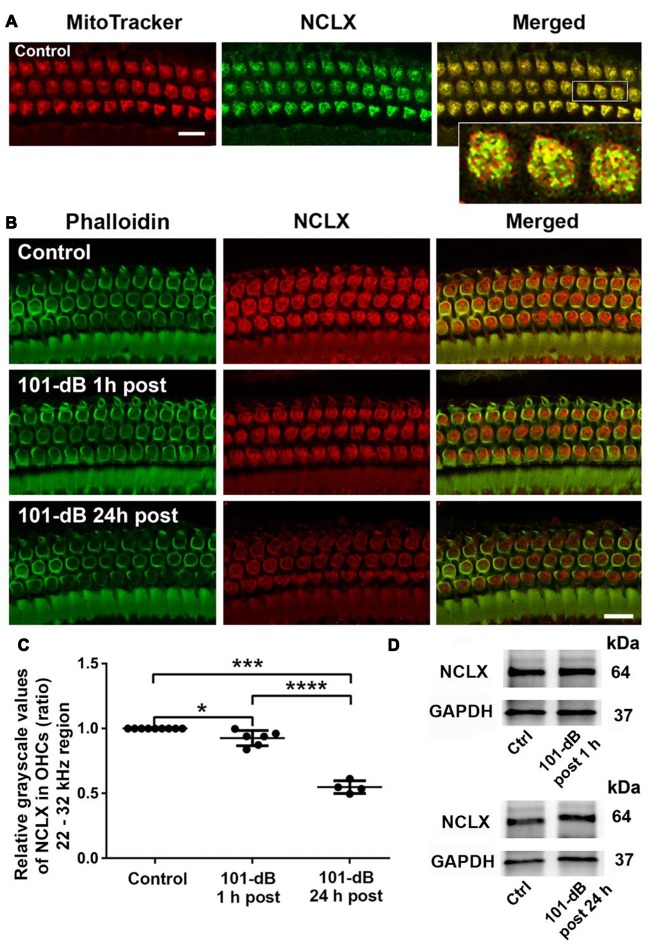
Noise exposure decreased NCLX immunoreactivity in OHCs of the basal turn in a time-dependent and intensity-dependent manner. **(A)** Representative images of surface preparations revealed co-localization of NCLX (green) and MitoTracker (red) in OHCs (merged, yellow). An enlarged image of three OHCs better illustrates the co-localization; scale bar = 10 μm, *n* = 3 per group with one cochlea used per mouse. **(B)** Representative images for NCLX in OHCs 1 h and 24 h after completion of the exposure. Green: phalloidin-stained OHCs. Images were taken from the 22–32 kHz region of the surface preparations using a Leica SP5 confocal microscope; scale bar = 10 μm. **(C)** Quantitative analysis of NCLX immunolabeling in OHCs showed a significant decrease in a time-dependent manner. Data are presented as individual points and means ± SD; *p < 0.05, ***p < 0.001, ****p < 0.0001. Control: *n* = 9, 101-dB 1 h post: *n* = 6, 101-dB 24 h post: *n* = 4 with one cochlea used per mouse. **(D)** Representative immunoblots of total cochlear homogenates from CBA/J mice showed no difference in NCLX band densities between control and noise exposed mice when examined 1 h and 24 h after completion of the exposure. GAPDH served as the sample loading control; *n* = 6 mice per group.

Since NCLX plays an important role in extrusion of mitochondrial calcium, we also assessed NCLX in NIHL. First, we tested the localization of the NCLX antibody to mitochondria by co-localization of MitoTracker with NCLX immunolabeling on surface preparations using control CBA/J mice (Figure 4A). Quantification of the overlap coefficient of MitoTracker and NCLX immunolabeling in OHCs revealed 95% overlap. We then conducted immunolabeling for NCLX with surface preparations under 101-dB conditions for three time points (control without noise exposure, 1 h post noise exposure, and 24 h post noise exposure). Immunolabeling for NCLX in OHCs of the basal turn corresponding to 22–32 kHz appeared weaker when processed 1 h after and was further reduced at 24 h after the completion of the noise exposure (Figure 4B). Quantification of immunolabeling for NCLX from original confocal images in OHCs confirmed a reduction both 1 h after and 24 h after the exposure with significantly greater reduction at 24 h (50% reduction) than 1 h (10% reduction) after the noise exposure (Figure 4C). Furthermore, we confirmed that noise-diminished NCLX immunolabeling in OHCs was reduced significantly more under 108-dB-noise-exposure conditions (30% reduction) than 101-dB conditions (10% reduction) when processed 1 h post noise exposure (Supplementary Figures S3A,B). Finally, we evaluated the specificity of the NCLX antibody by immunoblotting using whole cochlear homogenates. Western blots showed a single NCLX band at 64 kDa (Supplementary Figure S3C) without difference in the band density between mice exposed to noise at 101 dB or 108 dB SPL and un-exposed mice when processed 1 h after and 24 h after the completion of 101-dB noise exposure (Figure 4D). These results demonstrated that depression of NCLX immunoreactivity in the basal turn of OHCs by noise exposure is time- and noise-intensity-dependent, suggesting decreased extrusion of calcium from mitochondria in OHCs.

### Inhibition of Mitochondrial Calcium Uniporter in CBA/J Mice by Pretreatment With siRNA or Ru360 Reduces Noise-Induced Cleavage of Caspase 9 in the Basal Turn of Outer Hair Cells

**Figure 5:**
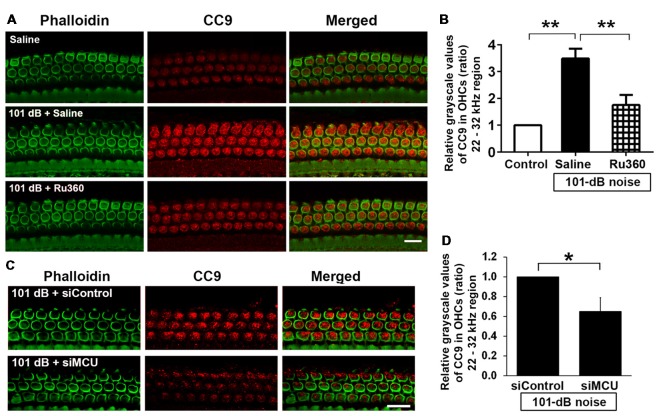
Inhibition of MCU *via* the pharmacological inhibitor Ru360 or siMCU attenuated noise-induced increases in cleaved caspase 9 (CC9) in OHCs of the basal turn. **(A)** Representative images show an increase in immunoreactivity for CC9 (red) in OHCs stained with phalloidin 1 h after completion of the exposure (panel 2) compared to control mice without exposure (panel 1). Treatment with Ru360 attenuated noise-induced CC9 in OHCs (panel 3). Images were taken from the region of the surface preparations corresponding to sensitivity to 22–32 kHz using a Leica SP5 confocal microscope; scale bar = 10 μm. **(B)** Quantification of CC9 in OHCs confirmed a significant increase after noise exposure and attenuation of this increase with Ru360 treatment; *n* = 4 per group with one cochlea used per mouse. Data are presented as means + SD, **p < 0.01. **(C)** Representative images show that pretreatment with siMCU decreases immunoreactivity for CC9 (red) in OHCs stained with phalloidin (green) 1 h after completion of the exposure compared to siControl treatment. Images were taken from the region of the surface preparations corresponding to sensitivity to 22–32 kHz using a Zeiss confocal microscope; scale bar = 10 μm. **(D)** Pretreatment with siMCU also significantly reduced noise-increased immunolabeling for CC9 in OHCs compared to mice exposed to scrambled siRNA (siControl). Data are presented as means + SD, *n* = 4 per group with one cochlea used per mouse, *p < 0.05.

Mitochondrial calcium overload initiates caspase-dependent cell death. Since noise exposure activates multiple cell death pathways, including apoptotic cell death (Zheng et al., 2014), we presumed that MCU inhibition diminishes mitochondrial calcium overload and should modulate apoptotic pathways. We inhibited MCU with the specific inhibitor Ru360 or with siRNA (siMCU). Based on the successful attenuation of noise-induced hair cell loss and hearing loss by treatment with Ru360 at 240 μg/kg, we used this dose for assessing inhibition of noise-activated CC9 in OHCs. According to our previously published results, CC9 was significantly elevated 1 h after noise exposure (Zheng et al., 2014); we therefore assessed CC9 at this time point. In agreement with our previous results, immunolabeling for CC9 in OHCs in the area of the basal turn corresponding to 22–32 kHz was significantly elevated after the noise exposure (Figures 5A,B). Such elevation of CC9 was significantly diminished by treatment with Ru360 (Figure 5B). Additionally, pretreatment with siMCU attenuated the reduction of CC9 immunoreactivity by 35% compared to noise-exposed mice 1 h after the noise exposure (Figures 5C,D).

### Impairment of Hearing at High Frequencies Is Found in Both MCU Knockout Mice and Their Wild-Type Littermates

**Figure 6:**
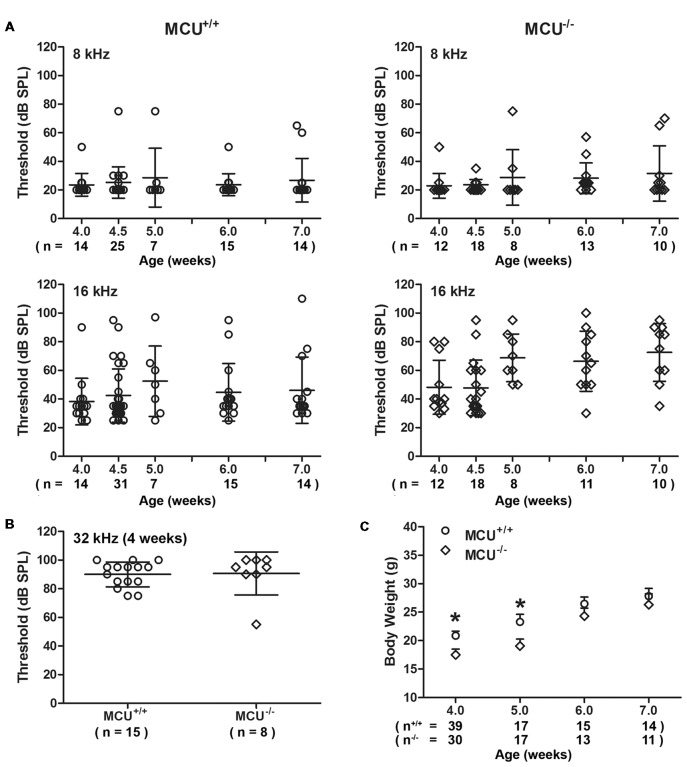
MCU knockout mice (MCU^−/−^) and wild-type littermates (MCU^+/+^) on a hybrid CD1 and C57/B6 background had OHC loss in the basal turn and high-frequency hearing loss. **(A)** Auditory brainstem response (ABR) thresholds at 8 kHz were not significantly elevated and not different between MCU^−/−^ mice and MCU^+/+^ from weeks 4 to 7 weeks of age but displayed wide variations at 16 kHz. Data are presented as individual points and means ± SD. **(B)** ABR thresholds at 32 kHz were greatly elevated in 4-week-old MCU^−/−^ and MCU^+/+^ mice without a significant difference between these two groups. Data are presented as individual points and means ± SD. **(C)** Body weights of MCU^−/−^ mice were significantly lower than that of littermates at the age of 4–5 weeks, but were the same as wild-type littermates by 6 weeks. Data are presented as individual points and means ± SEM, *p < 0.05. In panels **(A,B)** *n* indicates the number of mice per group with one cochlea used per mouse, *n* in panel **(C)** indicates the number of mice.

To further determine the role of MCU in NIHL, we used MCU knockout mice on an outbred C57BL/6 with CD1 background (Murphy et al., 2014). Since the CD1 strain has sensorineural hearing impairment at high frequencies (Le Calvez et al., 1998), we measured ABRs weekly from 4 weeks to 7 weeks of age for both MCU knockouts and wild-type littermates. Thresholds at 8 kHz remained around 25 dB SPL from 4 weeks to 7 weeks, with sporadic impairment in both MCU knockouts and wild-type littermates without significant differences between MCU knockouts and wild-type littermates (Figure 6A). However, there were elevations in auditory thresholds at 16 kHz in both MCU knockouts and wild-type littermates with wide variations between individual mice. For example, some mice maintained almost normal auditory thresholds, while others were completely deaf at this frequency (Figure 6A). Furthermore, baseline auditory thresholds at 32 kHz were already elevated to around 90 dB SPL at 4 weeks of age (Figure 6B). DAB-stained myosin-VIIa-labeled cochlear surface preparations of both MCU knockouts and wild-type littermates showed intact IHCs from the apex through the base; however, severe OHC loss appeared in the basal turn at 7 weeks of age (Supplementary Figure S4A), while OHCs in the apex appear normal by assays using both scanning electron-microscopy (Supplementary Figure S4B) and myosin-VIIa-labeled, DAB stained cochlear epithelia (Supplementary Figure S4A). Additionally, we observed that the body weights of MCU knockout mice were slightly lower than wild-type littermates at 4 and 5 weeks of age, and indistinguishable at 6 and 7 weeks of age (Figure 6C). These results indicate that knockout of the *MCU* gene alone did not alter auditory thresholds but highlight the fact that CD1 mice carry genetic hearing loss at high frequencies.

### MCU Knockout Mice Are More Resistant to Acoustic Stress Than Wild-Type Littermates

Since the majority of MCU knockout and wild-type littermates showed baseline auditory thresholds around 30 dB SPL at 8 kHz, we first exposed both MCU knockouts and wild-type littermates at the age of 4.5 weeks with baseline auditory thresholds of less than 30 dB SPL at 8 kHz to OBN centered at 4 kHz and intensities of 118 or 116 dB SPL for 2 h in order to set up appropriate noise exposure conditions. Surprisingly, sudden death occurred in the MCU wild-type littermates during the noise exposure, with a death rate of 40% (2/5) at 118 dB SPL and 7.5% (6/80) at 116 dB SPL. No MCU knockout mice (five knockout mice at 118 dB, 65 knockout mice at 116 dB SPL) died under either noise exposure condition, indicating that mice with knockout of the *MCU* gene are significantly resistant to general noise stress at 116 dB SPL (*p* = 0.033). We therefore chose the 116-dB SPL noise condition for further study. MCU knockouts and wild-type littermates were exposed to OBN at 116 dB SPL for 2 h at the age of 4.5 weeks. Two weeks after the noise exposure, permanent auditory threshold shifts at 8 kHz were noted in both MCU knockouts and wild-type littermates compared to non-noise-exposed age-matched control mice (Supplementary Figure S4C). MCU knockouts had average threshold shifts of 33 ± 8 dB, and those of wild-type littermates were 39 ± 11 dB with no statistical difference between these groups. Furthermore, this intensity of noise exposure did not induce OHC loss in the apex of the cochlear spiral. These results indicate that MCU knockouts were resistant to noise-induced seizures and noise exposure was unable to induce loss of OHCs in the apical region.

### MCU Knockout Mice Have Recovery of IHC Synaptic Ribbons After Noise Exposure and Attenuation of Noise-Diminished Wave I Amplitudes

**Figure 7:**
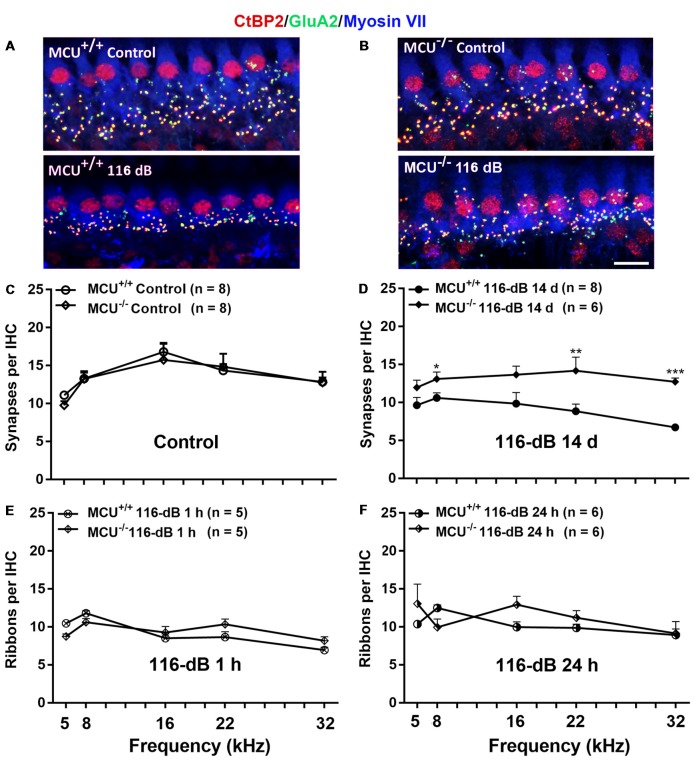
Noise-induced losses of synapses were attenuated in MCU knockout mice at 14 days after the completion of noise exposure. **(A,B)** Representative images of immunolabeling for IHC synapses in the apical region corresponding to 8 kHz of MCU^+/+^ or MCU^−/−^ mice examined 14 days after the noise exposure. The images were projected from Z sections. Red: CtBP2, green: GluA2, blue: myosin-VIIa-labeled IHCs; scale bar = 10 μm. **(C)** The number of synapses per IHC was similar between MCU^+/+^ and MCU^−/−^ mice without noise exposure. **(D)** The number of synapses had recovered significantly in MCU knockout mice but not in littermates when examined 14 days after the noise exposure. **(E)** Noise-induced loss of ribbons was also similar between MCU^−/−^ mice and littermates when examined 1 h after the completion of noise exposure. **(F)** Ribbons were not different between MCU^−/−^ mice and MCU^+/+^ when examined 24 h after the completion of noise exposure. Data are shown as means ± SEM in panels **(C–F)**, *n* indicates the number of mice with one cochlea used per mouse, *p < 0.05, **p < 0.01, ***p < 0.001.

Since IHCs are intact along the entire cochlear spiral of MCU knockouts and littermates, we determined if knockout of the *MCU* gene could attenuate noise-induced loss of synapses in IHCs. First, we compared the number of presynaptic ribbons and functional synapses in MCU knockouts and wild-type littermates without noise exposure 14 days after the completion of 116 dB SPL noise exposure. Functional synapses were assessed as juxtaposed presynaptic ribbons (CtBP2-labeled) and postsynaptic glutamate receptors (GluA2-labeled). Knockout of *MCU* alone did not impact the synapses Figures 7A,B, upper panels, and Figure 7C). After noise exposure, no obvious separation of the immunolabeling for CtBP2 and GluA2 was observed in either group (Figures 7A,B, lower panels). Additionally, some of the CtBP2 and GluA2 signals surrounded the nuclei or were even above the nuclei of IHCs in the MCU knockout mice regardless of noise exposure, but the majority of CtBP2 and GluA2 were located below the IHC nuclei (Figure 7B). A severe reduction in functional synapses was evident after noise exposure in wild-type mice from the apex to the base as measured at areas corresponding to 5, 8, 16, 22, and 32 kHz (for detailed statistical values see Supplementary Table S3). By contrast, MCU knockout mice had no significant loss of synapses 14 days after noise exposure compared to age-matched MCU knockouts without noise exposure. Further comparison of noise-exposed MCU knockouts to noise-exposed wild-type littermates showed that MCU knockouts were significantly resistant to noise-induced loss of synapses at the regions corresponding to 8, 22, and 32 kHz, but was not different in the 5- and 16-kHz regions (Figure 7D; for detailed statistical values see Supplementary Table S4). To further determine if the MCU knockouts are resistant to noise damage or have recovery of IHC ribbon synapses, we assessed ribbons at 1 h and 24 h after the completion of 116-dB noise exposure in both MCU knockouts and their littermates. Noise-induced loss of ribbons was not statistically different between groups when examined at 1 h and 24 h after the completion of exposure (Figures 7E,F). These results indicate that MCU knockouts had recovery of noise-induced loss of ribbon synapses.

**Figure 8:**
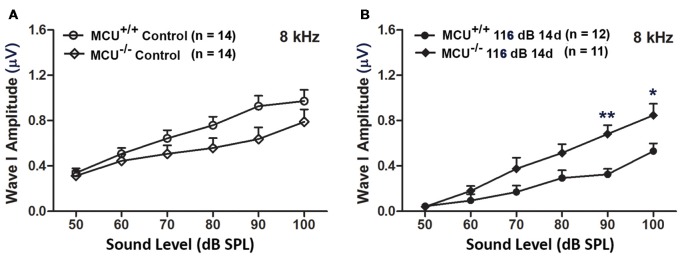
MCU knockout mice were resistant to reduction in their ABR wave I amplitudes at 8 kHz measured 14 days after noise exposure. **(A)** There was no significant difference in wave I amplitudes between MCU^−/−^ and MCU^+/+^ mice without noise exposure. **(B)** MCU knockouts were significantly resistant to reduction in ABR wave I amplitudes after noise in comparison to wild-type littermates at sound intensities of 90 and 100 dB SPL. Data are shown as means ± SEM, *p < 0.05, **p < 0.01. *n* indicates the number of mice; one cochlea was used per mouse.

In addition, we evaluated ABR wave I amplitudes (8 kHz) as functional markers for synaptic integrity. Knockout of the *MCU* gene alone (without noise exposure) did not alter wave I amplitudes (Figure 8A). In agreement with our previous reports and those of others (Kujawa and Liberman, 2009; Wan et al., 2014; Hill et al., 2016), noise exposure reduced wave I amplitudes in wild-type littermates at sound stimulation levels from 50 dB to 100 dB SPL (for detailed statistical values see Supplementary Table S5). By contrast, noise exposure did not significantly reduce wave I amplitude levels in MCU knockout mice at sound stimulation levels of 90 dB and 100 dB SPL (Figure 8B).

## Discussion

The salient finding of this study is that inhibition of MCU *via* pretreatment with MCU siRNA or the selective MCU inhibitor Ru360 reduced PTS-noise-induced losses of IHC synaptic ribbons, OHCs, and wave I amplitudes, as well as NIHL in adult CBA/J mice. Finally, MCU knockout mice with intact IHCs are resistant to noise-induced seizures and have the capacity for recovery of IHC synapses after extremely high noise exposure.

### Mitochondrial Transporters Are Important Modulators of Noise-Induced Cochlear Synaptopathy, Hair Cell Death, and Functional Deficits in CBA/J Mice

While mitochondrial calcium uptake by MCU under stress is a means to maintain cytosolic calcium homeostasis (Boitier et al., 1999; Kirichok et al., 2004; Chaudhuri et al., 2013), it may lead to pathologically high mitochondrial calcium, triggering cell death (Mattson, 2007; Celsi et al., 2009). The hypothesis that excessive mitochondrial calcium due to increased MCU expression and decreased NCLX contributes to OHC death is clearly supported by the reduction of noise-induced OHC loss and protection against NIHL after treatment with siMCU or the selective inhibitor Ru360 in CBA/J mice. In addition to protecting hair cells, MCU inhibition reduced the extent of noise-induced IHC synaptic ribbon loss, thereby preventing the decline of ABR peak I amplitudes. These results implicate MCU as an important mediator affecting cochlear synaptopathy, hair cell death, and functional deficits. Such an action is in line with previous reports in cortical and hippocampal neurons in which the inhibition of MCU *via* knockdown exerted a neuroprotective effect from NMDA-induced excitotoxicity (Qiu et al., 2013) and a recent report on transcriptional repression of MCU reducing excitotoxicity (Depp et al., 2018). Likewise, Ru360 treatment has also been shown to reduce pathological mitochondrial calcium uptake in various cell types, including cochlear supporting cells such as Claudius’ and Deiters’ cells of neonatal rat cochlear explants (Mann et al., 2009). It is interesting that reduction of MCU by local intra-tympanic application of siMCU prior to noise exposure showed similar effects as that of Ru360 treatment. We believe that Ru360 treatment only partially inhibits MCU channel function in CBA/J mice. Likewise, treatment with siMCU also decreases MCU in OHCs. Such a decrease might also partially influence MCU function. However, the detailed mechanisms on how reduction of MCU expression decreases channel function needs further investigation.

The downstream mechanisms by which MCU can protect against hair cell loss remain speculative. Mitochondrial calcium overload and over production of reactive oxygen species (ROS) have been associated with necrotic and apoptotic cell death pathways *via* a sustained opening of the mitochondrial permeability transition pore (mPTP), resulting in collapse of mitochondrial membrane potential, and release of cytochrome C (Szalai et al., 1999; Nicotera et al., 2003; Rizzuto et al., 2012). Conversely, blockade of the mPTP, reduction of mitochondrial calcium uptake and inhibition of ROS production can serve a protective role against mitochondria-mediated cell death in neuronal cells and in NIHL (Baines et al., 2005; Sha and Schacht, 2017). Supporting such a concept for noise trauma, our current results show inhibition of MCU *via* siMCU or Ru360 treatment significantly reduced the CC9 in OHCs, an essential downstream step in the activation of intrinsic mitochondria-dependent apoptotic pathways. Still, treatment with Ru360 was insufficient to protect from severe PTS-NIHL (108-dB, SPL) in CBA/J mice, suggesting that such a high-level exposure may trigger additional cell death pathways and that pharmacological protection must be directed at multiple targets. Of note, our previous report showed that inhibition of noise-induced apoptosis in OHCs shifts the predominant cell death pathway to necrosis under such severe PTS noise exposure (Zheng et al., 2014).

Noise exposure, especially higher intensities, decreases capillary blood flow and causes local vasoconstriction, resulting in ischemia (Quirk and Seidman, 1995; Miller et al., 2003). Such ischemia depletes ATP levels within inner ear structures, including sensory hair cells and the stria vascularis (Nagashima et al., 2011; Chen F.-Q. et al., 2012). In support of transient cellular ATP depletion in sensory hair cells, a homeostatic energy sensor, adenosine monophosphate-activated protein kinase (AMPK), is activated after noise exposure known to induce permanent hearing loss (Hill et al., 2016). Noise exposure, especially higher intensities, triggers elevation of intracellular Ca^2+^ levels, followed by rapid Ca^2+^ entry into the mitochondrial matrix *via* MCU (Chen Q. et al., 2012). The enhanced MCU function following noise exposure may initially be an adaptive response to stress, trapping excessive amounts of intracellular Ca^2+^ in mitochondria to maintain Ca^2+^ homeostasis in the cytosol, while the initial increased mitochondrial calcium levels stimulate mitochondria to generate ATP (Clapham, 2007). However, prolonged Ca^2+^ uptake eventually leads to mitochondrial Ca^2+^ overload, resulting in collapse of the mitochondrial membrane potential, uncoupling of oxidative phosphorylation in the respiratory chain, and over-production of ROS. It is well known that oxidative imbalance contributes to sensory hair cell death after inner ear trauma including noise-induced, ototoxic drug-induced, and age-related hearing loss (Jiang et al., 2007; Oishi and Schacht, 2011; Chen et al., 2013). While MCU controls uptake of mitochondrial calcium, extrusion of calcium from mitochondria is mediated primarily by a mitochondrial sodium calcium exchanger (NCLX), encoded by the *NCLX* gene (Palty et al., 2012). Like MCU, NCLX is localized to the mitochondrial inner membrane, where it regulates mitochondrial calcium concentrations and shapes intracellular calcium signaling. Under normal physiological conditions, mitochondrial calcium uptake and release are in equilibrium. NCLX has been shown to be involved in neuronal death in a model of Parkinson disease (Gandhi et al., 2009; Palty et al., 2012). Noise exposure decreased NCLX expression in OHCs in a time- and intensity-dependent manner, indicating alteration of NCLX function. Although the detailed mechanisms of decreased NCLX function in sensory hair cells after noise exposure need to be investigated further, the noise-induced decrease in NCLX in OHCs seems to act as a deleterious complement to the increase in MCU and further augments mitochondrial calcium overload.

In addition, we need to review our negative results by Western analysis. Noise-induced loss of sensory hair cells in mice does not map to the frequencies of noise exposure. Regardless of whether mice are exposed to the OBN (8–16 kHz) or broadband noise (2–20 kHz), noise-induced loss of sensory hair cells follows a base-to-apex gradient with losses of sensory hair cells beginning at the basal turn of the cochlear spiral and the changes in molecular signals in OHCs also showed a similar pattern (Yuan et al., 2015; Hill et al., 2016). Due to the limitation of mouse cochlear tissues by Western blot analysis being assessed in whole cochlear homogenates that contain all three turns of the cochlear spiral (apex, middle, and basal turn) and multiple cochlear cell types, changes in specific regions are muted when assessed by Western blot. Therefore, changes in MCU or NCLX only in the cochlear sensory hair cells of the basal turn might be diluted by other cochlear cell types resulting in unchanged total MCU or NCLX by Western blot.

### Noise-Induced Loss of Inner Hair Cell Synapses Is Reversed in MCU Knockout Mice

The fact that MCU knockout mice are viable indicates compensatory or alternative pathways for the entry of mitochondrial calcium. The scientists who originally generated MCU knockout mice reported that mitochondrial calcium levels are reduced but not absent in the MCU knockout mice (Murphy et al., 2014). This supports the studies from another group showing that other MCU-independent mitochondrial calcium channels, such as transient receptor potential channels, are also responsible for mitochondrial calcium uptake (Feng et al., 2013).

The observed basic hearing characteristics of MCU knockout and wild-type littermates are in agreement with what is known of the well-documented CD1 strain, which has sensorineural hearing loss at high frequencies correlating to the basal turn (Le Calvez et al., 1998), but not at low frequencies, such as 8 kHz, and OHCs at apical turn remain intact. Furthermore, IHCs also remain intact along the entire cochlear spiral. Based on this feature, high-intensity noise exposure (116-dB SPL OBN centered at 4 kHz) was imposed on MCU knockout and wild-type littermates in order to induce OHC loss at the apex; this is the highest noise intensity that MCU wild-type mice survived. Unfortunately, such high noise intensity is unable to induce OHC loss at the apex, although we observe moderate hearing impairment at 8 kHz 14 days after the noise exposure. The mammalian cochlea has a tonotopic organization producing an exponential frequency map (Müller et al., 2005). The different vulnerability of OHCs to inner ear damage is a well-documented phenomenon, although the detailed mechanism is unknown (Sha and Schacht, 2017). It has been suggested that the ability of plasma membrane calcium ATPase (PMCA2) to extrude calcium load through mechanotransducer channels is limited in the OHC basal turn, causing cytosolic calcium overload and leading to high-frequency hearing loss (Chen Q. et al., 2012). Additionally, OHCs in the basal turn are more susceptible to free radical damage (Sha et al., 2001). Furthermore, stiffness of the basilar membrane is different between base and apex (Liu et al., 2015). Recently, it has been suggested that there is a high-pass filter at the cochlear apex, where the mechanical turning curves are less aligned with the nerve fiber tuning curves (Fettiplace, 2017).

Since IHCs are intact along the whole cochlear spiral, we have focused our investigation on noise-induced changes in ribbon synapses using MCU knockouts and wild-type littermates. In line with our observations in CBA/J mice, high-intensity noise exposure induces loss of ribbon synapses in MCU wild-type littermates without significant recovery by 14 days after the exposure (Hill et al., 2016). However, MCU knockouts suffer only temporary damage rather than permanent noise-induced IHC synapse loss, allowing IHC synapses to fully recover and protect against the decline of ABR wave I amplitudes. These results are compatible with our data from treatment with siMCU and Ru360 showing protection against noise-induced loss of ribbon synapses in CBA/J mice, indicating MCU as an important mediator affecting cochlear synaptopathy. In the current study, there is no evidence of retraction of peripheral nerve endings from the CtBP2 puncta when examined 14 days after the exposure. While such retraction is reported in the literature (Shi et al., 2013; Liberman et al., 2015), the majority of CtBP2 puncta were co-localized with GluA2. The CtBP2 and GluA2 signals surrounding or above the nuclei of IHC in the MCU knockout mice cannot be attributed to an effect of noise exposure, as such scenario occurred regardless of noise exposure. Additionally, the majority of CtBP2 and GluA2 were located below the IHC nuclei. The detailed downstream mechanisms by which knockout of the *MCU* gene can protect against IHC synapse loss and the plasticity of ribbon synapses after noise damage require further investigation. As we have discussed above, noise-induced mitochondrial calcium overload may lead to overproduction of ROS, which is associated with noise-induced ribbon loss (Fetoni et al., 2013). Furthermore, recovery of noise-induced loss of ribbon synapses in MCU knockout mice is in line with the excitotoxicity theory, as transcriptional repression of MCU reduces excitotoxicity (Depp et al., 2018).

In summary, our results establish for the first time that noise-induced elevation of MCU and reduction of NCLX immunoreactivity in sensory hair cells may facilitate NIHL by mediating the loss of IHC synaptic ribbons and OHCs. The upregulation of MCU and decrease in NCLX in OHCs of the basal turn after noise exposure is most likely a response to increased intracellular calcium levels. Agents that inhibit MCU activity reduce the extent of mitochondrial calcium overload and, subsequently, decrease the induction of apoptotic pathways associated with hair cell loss.

## Author Contributions

XW, HL, HX, RL, KH and HY performed research in CBA/J mice. YZ performed research in MCU KO mice. SP and QF performed research in CBA/J and MCU KO mice. KH performed research in OC-1 cells. S-HS designed research, analyzed the data and wrote the article. All authors have reviewed the contents of the manuscript, approve of its contents, and validate the accuracy of the data.

## Conflict of Interest Statement

The authors declare that the research was conducted in the absence of any commercial or financial relationships that could be construed as a potential conflict of interest.

## Abbreviations

ABR: auditory brainstem response
CaM: Ca^2+^-binding protein calmodulin
CC9: cleaved caspase 9
DAB: diaminobenzidine
EDTA: ethylenediaminetetraacetic acid
IHC: inner hair cell
MCU: mitochondrial calcium uniporter
mPTP: mitochondrial permeability transition pore
NCLX: sodium calcium exchanger
NIHL: noise-induced hearing loss
OBN: octave band noise
OC: organ of Corti
OHC: outer hair cell
PBS: phosphate buffered saline
PNH1: post noise exposure 1 h
PNH24: post noise exposure 24 h
PTS: permanent threshold shift
RIPA: radioimmunoprecipitation assay
RWN: round window niche
SGN: spiral ganglion neuron
SPL: sound pressure level.
