# Supplementary material for: Mitochondrial Calcium Transporters Mediate Sensitivity to Noise-Induced Losses of Hair Cells and Cochlear Synapses
Source: Front Mol Neurosci. 2019 Jan 8;11:469. doi: 10.3389/fnmol.2018.00469 (PMC6331433; doi:10.3389/fnmol.2018.00469)
Supplement: Supplementary file 1 [file Data_Sheet_1.pdf]

Supp. Figure 1

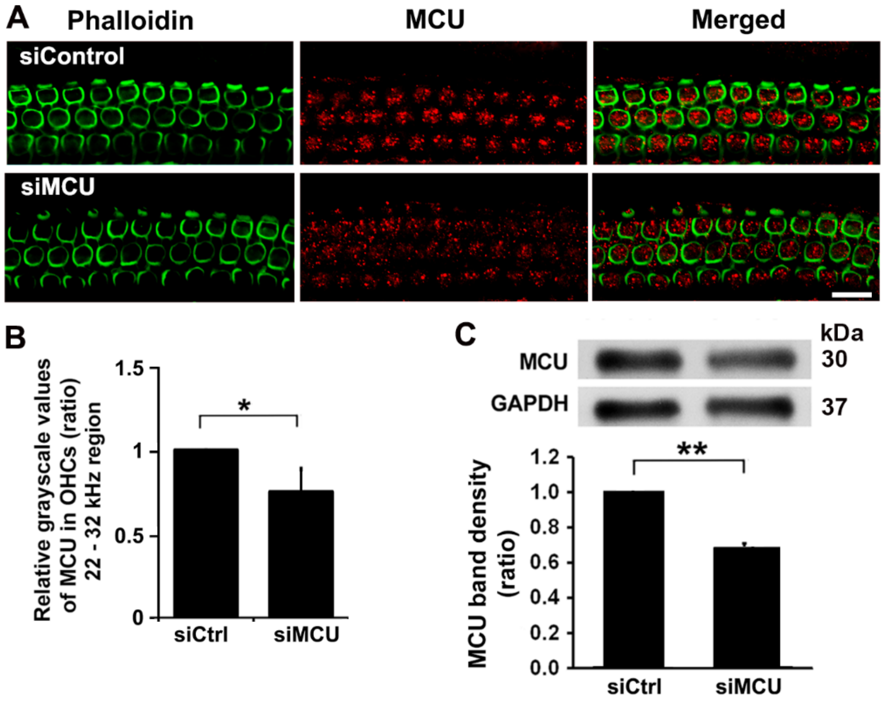

Supp. Figure 1. Inhibition of MCU via siMCU pretreatment reduces MCU expression in cochlear epithelia of CBA/J mice and 3T3 cells. (A) Representative images show that pretreatment with siMCU for 72 h decreases immunoreactivity for MCU (red) in OHCs stained with phalloidin (green) compared to siControl treatment. Images were taken from the region of the surface preparations corresponding to sensitivity to 32 kHz using a Zeiss confocal microscope, scale bar = 10 µm. (B) Quantification of MCU immunolabeling in OHCs confirmed significant reduction after siMCU treatment. Data are presented as means + SD, $n = 4$ per group with one cochlea used per mouse, $*p < 0.05$. (C) Immunoblots using formalin-fixed cochlear epithelium homogenates showed reduction of MCU expression 72 h after MCU siRNA treatment compared to scrambled siRNA (siCtrl) in CBA/J mice. Data are shown as means ± SD. Each sample was pooled from 4 mice, three samples per group; GAPDH served as the sample loading control, $**p < 0.01$.

Supp. Figure 2

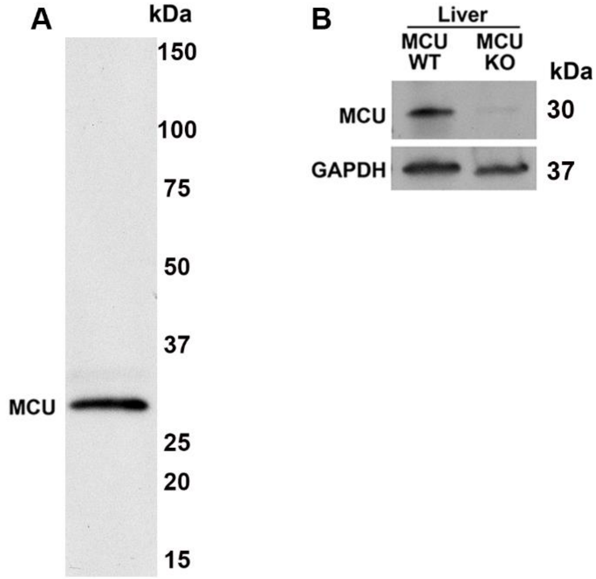

Supp. Figure 2. Immunoblots show specificity of the MCU antibody. (A) A representative Western blot reveals the specificity of the MCU antibody at the molecular weight of 30 kDa in cochlear HEI-OC-1 cell homogenates. (B) Liver homogenates of MCU wild-type mice showed a band corresponding to MCU, which was absent in MCU knockout mice. GAPDH served as the sample loading control.

Supp. Figure. 3.

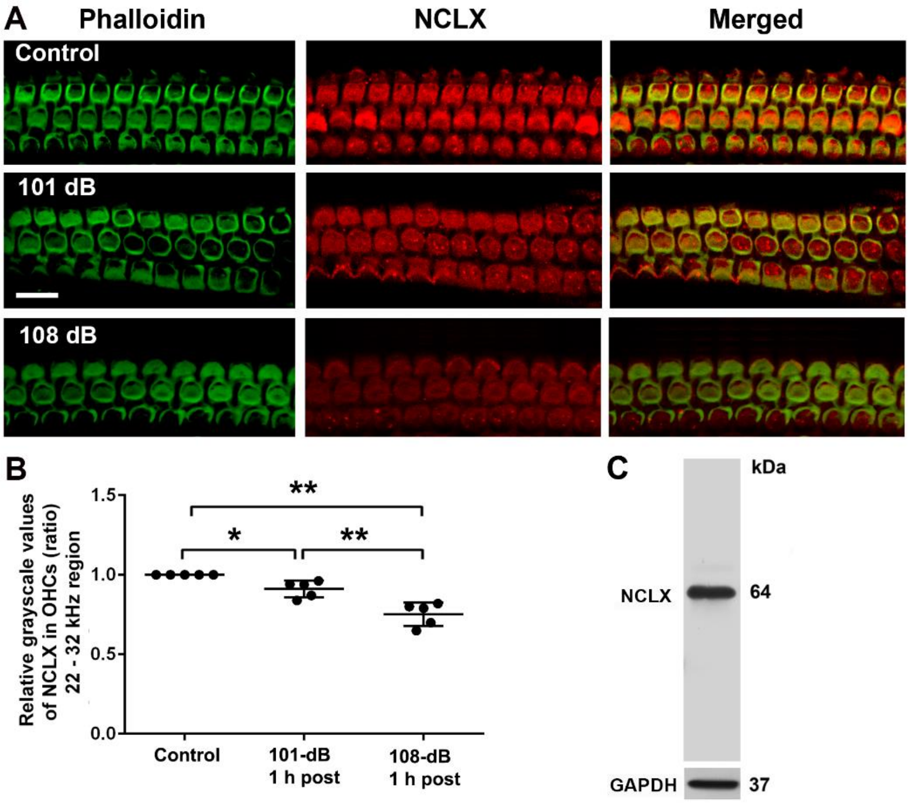

Supp. Figure 3. Decreased-NCLX immunolabeling in OHCs of the basal turn is also noise-intensity dependent. (A) Representative images of NCLX immunolabeling in OHCs showed a greater decrease after 108-dB compared to 101-dB exposure examined 1 h after the completion of the noise exposure. Images were taken from the region of the surface preparations corresponding to sensitivity to 22-32 kHz using a Zeiss confocal microscope; scale bar = 10 µm. (B) Quantification of NCLX immunolabeling confirmed a significant decrease. Data are presented as individual points and means ± SD; $n = 5$ mice per group with one cochlea used per mouse, *$p < 0.05$, **$p < 0.01$. (C) Immunoblots of whole cochlear homogenates from control CBA/J mice showed a single NCLX band at 64 kDa. GAPDH served as the sample loading control.

Supp. Figure 4

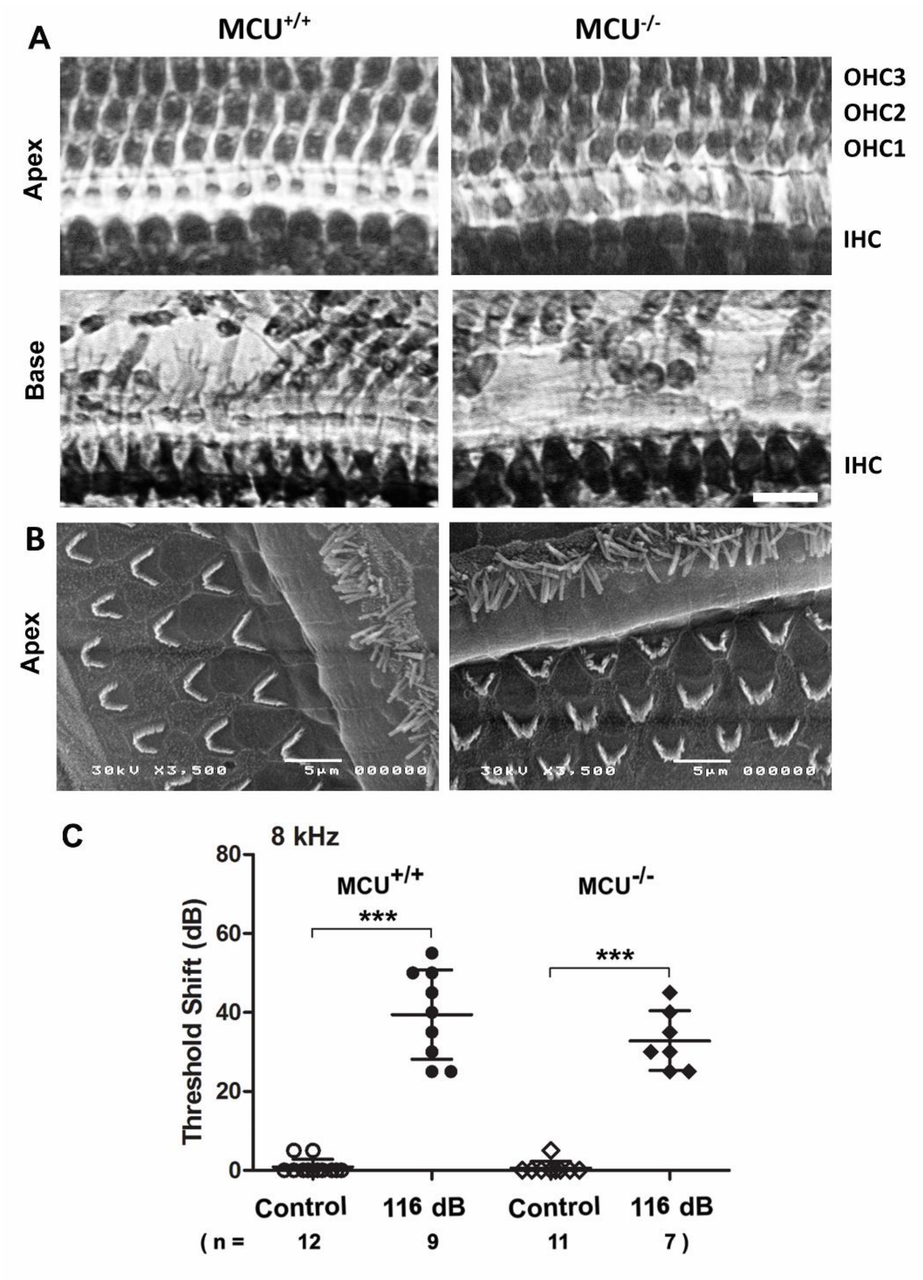

Supp. Figure 4. Both MCU knockout mice and wild-type littermates show loss of OHCs in the basal turn, but IHCs are intact at 7 weeks of age. (A) Representative images revealed almost complete loss of OHCs in the basal turn in both the $MCU^{-/-}$ and $MCU^{+/+}$, while OHCs in the apex remained intact. All of the IHCs are intact. OHC1, 2, and 3 indicate the three rows of OHCs and IHC indicates inner hair cells; scale bar = 10 µm. These images represent 6 mice per group using

both ears. (B) Scanning electron microscopy revealed that OHC stereocilia of MCU knockout mice and wild-type littermates were intact in the apical region. Scale bars = 5 μm. These images represent the left ears of 3 mice per group. (C) Noise-exposure at 116 dB SPL significantly increased auditory threshold shifts at 8 kHz in both $MCU^{-/-}$ and $MCU^{+/+}$, but no significant difference between the two groups is seen when examined 14 d after the noise exposure. Data are presented as individual points and means ± SD; ***$p < 0.001$, n indicates the number of mice per group with one cochlea used per mouse.

Supp. Table 1: Post-hoc testing for data in figure 2B (synaptic ribbons/IHC in CBA/J mice)

Table 1: Post-hoc			
**Noise (dB SPL)**	**Groups**	**Frequency (kHz)**	***p* value**
101	Control vs 101 dB	5	*p* = 0.2554
		8	*p* < 0.001
		16	*P = 0.0007*
		22	*p* < 0.001
		32	*p* = 0.0041

Supp. Table 2: Post-hoc testing for data in figure 2C (ABR wave I amplitude in CBA/J mice)

Table 2: Post-hoc			
**Frequency (kHz)**	**Groups**	**Sound Intensity**	***p* value**
16	Control vs 101 dB	30	*P* < 0.0001
		40	*P* < 0.0001
		50	*p* < 0.0001
		60	*p* < 0.0001
		70	*p* < 0.0001
		80	*p* < 0.0001
		90	*p* < 0.0001
		100	*p* < 0.0001
	Saline vs Ru306	60	*p* = 0.3100
		70	*p* = 0.0333
		80	*P < 0.0001*
		90	*p* < 0.0001
		100	*p* < 0.0001
	101 dB + Saline vs 101 dB + Ru360vs	60	*p* = 0.4933
		70	*p* = 0.1444
		80	*p* = 0.0255
		90	*p* < 0.0001
		100	*p* < 0.0001

Supp. Table 3. *Post hoc* testing for data of synapses/IHC in MCU wild-type littermates

Group	Frequency (kHz)	*p* values
MCU$^{+/+}$ Control vs. *MCU*$^{+/+}$ 116 dB 14d	5	0.254
	8	0.001
	16	0.004
	22	0.144
	32	0.012

Supp. Table 4. *Post hoc* testing for data in figure 8F (synapses/IHC in MCU mice)

Group	Frequency (kHz)	*p* values
MCU$^{+/+}$ 116 dB 14d vs. *MCU*$^{-/-}$ 116 dB 14d	5	0.014
	8	0.000
	16	0.143
	22	0.011
	32	0.000

Supp. Table 5. *Post hoc* testing for data of wave I amplitudes in wild-type MCU littermates

Group	Sound level (dB SPL)	*p* values
MCU$^{+/+}$ Control vs. *MCU*$^{+/+}$ 116 dB 14d	50	0.000
	60	0.000
	70	0.000
	80	0.000
	90	0.000
	100	0.002
